# Liquid Embolic Agents for Endovascular Embolization: A Review

**DOI:** 10.3390/gels9050378

**Published:** 2023-05-04

**Authors:** Amrita Pal, Jeffrey Blanzy, Karime Jocelyn Rosas Gómez, Mark C. Preul, Brent L. Vernon

**Affiliations:** 1 Center for Interventional Biomaterials, School of Biological and Health Systems Engineering, Arizona State University, Tempe, AZ 85287, USA; 2 The Loyal and Edith Davis Neurosurgical Research Laboratory, Department of Neurosurgery, Barrow Neurological Institute, Phoenix, AZ 85013, USA

**Keywords:** liquid embolic agents, gel, embolization

## Abstract

Endovascular embolization (EE) has been used for the treatment of blood vessel abnormalities, including aneurysms, AVMs, tumors, etc. The aim of this process is to occlude the affected vessel using biocompatible embolic agents. Two types of embolic agents, solid and liquid, are used for endovascular embolization. Liquid embolic agents are usually injectable and delivered into the vascular malformation sites using a catheter guided by X-ray imaging (i.e., angiography). After injection, the liquid embolic agent transforms into a solid implant in situ based on a variety of mechanisms, including polymerization, precipitation, and cross-linking, through ionic or thermal process. Until now, several polymers have been designed successfully for the development of liquid embolic agents. Both natural and synthetic polymers have been used for this purpose. In this review, we discuss embolization procedures with liquid embolic agents in different clinical applications, as well as in pre-clinical research studies.

## 1. Introduction

Endovascular embolization (EE) is a minimally invasive surgical procedure for the treatment of blood vessel abnormalities in various regions of the body. Endovascular treatment decreases hospitalization time and speeds up recovery as compared to open surgery [1]. EE can be used as a sole form of treatment or combined with a presurgical procedure targeted at augmenting blood flow, structure, or pathology. This allows a safer and more effective surgical treatment. EE is performed for the treatment of clinical conditions including aneurysms, arteriovenous malformations (AVMs), tumors, etc. The therapeutic goal of endovascular embolization is the complete occlusion of the affected vessel, reduction of blood flow, and the reduction of the neurovascular disease-related risks and symptoms utilizing an injected or positioned material [2]. Therefore, the safety and effectiveness, biocompatibility, biodegradability, and biomechanical properties of the embolic agents utilized, and optimizing technical aspects of the embolization procedures, are crucial [3]. High selectivity and accuracy, as well as avoidance of undesired events, including vessel perforation, embolization of normal vessels, and reflux, are necessary to perform effective EE procedures. During embolization, embolic substances, including metal coils, tiny spheres, chemicals, and glue, are used to eliminate the vessel or to obstruct blood flow.

Liquid embolic agents are usually injectable and delivered into the vascular malformation sites using a catheter guided by X-ray imaging (i.e., angiography). After the injection, the liquid embolic agents transform into a solid implant in situ, based on a variety of mechanisms, including polymerization, precipitation, and cross-linking, through ionic or thermal processes [3]. The biomaterial characteristics of the liquid embolic agents, including viscosity, solidification time, etc., play an important role in the whole embolization process, starting with the catheter access (diameter: 0.2–3.0 mm, length: up to 200 cm) to the occlusion of the target site by thrombosis or direct obstruction of the lesion [3]. Visibility is a critical safe delivery aspect of liquid embolic agents at the target site and can be achieved by adding several radiopaque additives or using different radiopaque solvents [3].

Embolic materials are classified into solid embolic agents or liquid embolic agents. Solid embolic agents include metal coils and plugs, which are mostly used for the treatment of focal vascular abnormalities [4]. Studies on solid embolic agents are not included here as they are not within the scope of this review. Liquid embolic agents include particulates, polymers, or in situ gelling biomaterials, which are used for the treatment of diffuse vascular abnormalities and are usually deliverable via catheters [4]. Liquid embolic agents can be classified as permanent or temporary. Temporary embolic agents are fast-acting and used for occluding a hemorrhaging vessel, whereas permanent embolic agents are long-lasting and used for the treatment of complex vascular malformation [4]. Until now, several polymers have been designed successfully for the development of liquid embolic agents [5]. Both natural and synthetic polymers have been used for this purpose [6]. In this review, we will discuss several liquid embolic agents used for endovascular embolization in different clinical applications as well as in pre-clinical research studies.

## 2. Clinical Applications of Liquid Embolic Agents

### 2.1. High-Flow Vascular Malformations

High-flow vascular malformations occur when blood flows from a feeding artery directly to a draining vein, bypassing the capillary bed [7]. Because of their high flow, these vascular malformations cause significant rerouting or shunting of blood flow away from surrounding tissue. The two main types of high-flow vascular malformations are known as arteriovenous malformations (AVMs) and arteriovenous fistulas (AVFs) [7]. AVMs are composed of abnormal arteries and veins, with blood shunting occurring through a central collection of dysmorphic vessels (i.e., the nidus) [7]. In an AVF, blood shunting occurs through a single arterialized vein instead of a nidus and is more commonly seen in the central nervous system [8]. The absence of capillaries results in high-pressure blood flow into veins, causing them to widen and often leads to rupture of the vessel. AVMs should be treated quickly to prevent risk of hemorrhage, stroke, heart-attack, neurological deficit, or seizure [4,9]. Cerebral AVMs may cause morbidity and neurological deficit and present a risk of hemorrhaging [10]. Both types of vascular malformations present similar challenges in treatment [7,11], often requiring several embolization procedures (e.g., transarterial embolization, transvenous embolization, or direct puncture embolization), sometimes combined with surgery as the primary treatment option [12].

The goal of endovascular embolization of AVMs is the closure of the whole nidus without occluding other surrounding normal and important vessels [2,13]. Although surgical resection is recommended for the management of AVMs, pre-surgical embolization aims to ease surgical removal, decrease surgery complications, and reduce blood loss during surgery [2,13]. Successful penetration into the nidus and draining vein requires the microcatheter to be positioned less than 1 cm from the nidus, which often cannot be achieved [14]. In such cases, occlusion of the arterial supply is sufficient if the AVM is surgically accessible for subsequent resection [11,13,15]. Incomplete resection or embolization may lead to further angiogenesis, which could increase the angioarchitecture complexity, thus making its subsequent treatment more challenging [12]. For example, angiogenesis may manifest as the creation of new feeders that may be too narrow to allow microcatheter access in subsequent treatment [12].

AVFs remain challenging when embolization is not accompanied by resection in that their high-flow nature may cause embolic materials to migrate into the distal draining veins, potentially resulting in unintentional pulmonary embolization [11,12]. Similarly, the reflux of liquid embolics into the distal venous drainage or proximal arteries during embolization is often difficult to control and should be prevented. Surgical resection is necessary if venous drainage is observed [12,16]. Reports of successful AVF treatments by endovascular embolization include dural, brain, and scalp arteriovenous fistulas. Dural arteriovenous fistulas (dAVFs) within the dura mater of the brain represent 10–15% of all intracranial AVMs [15,17]. They may present disabling symptoms, and hemorrhage occurs in about 65% of patients [17]. Congenital brain AVFs account for 1.6–4.7% of all brain AVMs and are characterized by the absence of a nidus and their high-flow nature [11]. Although they can be asymptomatic, they can also produce seizures, hemorrhage, and increased and intracranial pressure, among other symptoms that make treatment necessary [10,11]. The large draining vein can interfere with the exposure of the fistula, making surgery challenging and not ideal [11]. Endovascular management facilitates localization of the lesion and enables access to deep and/or critical areas [11]. The heterogeneous angioarchitecture and non-uniform structure of scalp AVFs makes them challenging to treat [12]. Their high-flow shunting can lead to blood loss if the fistula is punctured during resection, for which endovascular management is generally recommended [12].

### 2.2. Hypervascular Tumors

Hypervascular tumors consist of abnormally large numbers of blood vessels feeding into or contained within the tumor [4]. These tumors are commonly found in the head and neck. Examples include meningiomas, paragangliomas, and hepatocellular and colorectal carcinoma metastases [4]. High-grade gliomas, such as glioblastoma multiforme, may also have significant hypervascular composition. The higher blood flow in the blood vessels increases the risk of bleeding that may create a difficult surgical resection. Presurgical endovascular embolization of hypervascular tumors has proven to mitigate blood loss, operating time, and infection rates from surgical resection [18,19], and may decrease surgical morbidity and mortality [20]. The cost may be high for preoperative embolization of hypervascular tumors due to the large volume required for complete devascularization [18]. However, the advantages of preoperative embolization, in addition to the low complication rate, have increased its preference [18]. For example, anterior skull-based meningiomas fed by the ophthalmic artery may benefit from embolization by reducing the risk of visual impairment [20]. Usually, devascularization is performed using polyvinyl alcohol (PVA) microparticles [19]. When the feeders to the metastatic region also supply the anterior spinal artery, e.g., spinal hypervascular tumors, there is a risk that microparticles will migrate, posing a high risk of neurological complication [19]. When the vessels are small and tortuous, they may not be accessible by micro-catheterization [19]. In such cases, if surgically accessible, direct puncture of the lesion is recommended to administer microparticles or liquid embolic agents [19]. Alternatively, injection of Onyx, a widely used liquid embolic agent, by an endovascular route or by direct puncture has been used to successfully devascularize hypervascular tumors while providing a low risk of uncontrolled migration [19].

### 2.3. Aneurysms

Aneurysms are dilatations or bulges caused in weakened vessel walls, which can rupture and cause internal bleeding if the tension on the weakened wall increases [4]. Aneurysms can occur throughout the body, but are more common in the brain, aorta, renal artery, legs, spleen, and AVMs. It is estimated that 6.7 million people in the United States have one or more unruptured brain aneurysms each year, 10% of which need treatment [21]. About 30,000 people in the U.S. suffer an aneurysm rupture each year, which can result in death in 50% of cases, or permanent neurological deficit in 66% of those who survive [21]. The objective of treating aneurysms is their complete and permanent occlusion [22]. Ideally, this would be accompanied by the remodeling of the parent artery by endovascular embolization with an embolic agent. However, the use of platinum coils to treat large, wide-necked intracranial aneurysms often requires repeat treatment due to coil compaction, as well as the use of a stent to prevent migration to nearby vessels [22,23]. Liquid embolic agents achieve homogeneous and complete filling of aneurysms as opposed to coils. Furthermore, infectious aneurysms are often irregular in shape, making endovascular embolization with liquid embolic agents ideal [24].

The most common complications after endovascular aneurysm repair are type I and type II endoleaks, characterized by the persistent perfusion within the aneurysmal sac. Type I endoleaks result from an inadequate seal and can be seen either at the proximal end of the graft (type Ia) or at the distal end of the graft (type Ib) [25]. The occurrence of type I endoleaks are reported in the range of 2.9–6.9%, whereas type II endoleaks occur more frequently, ranging between 10–45% of all EVAR processes [26]. Type I endoleaks require immediate treatment to prohibit the risk of sac rupture, whereas type II endoleaks are correlated with sac reperfusion caused by collateral vessels. About 40–58% of cases are not associated with sac enlargement and resolve spontaneously [26]. Although transcatheter embolization is generally accepted as the primary treatment option for type II endoleaks, it has not been popular for the treatment of type I endoleaks [25]. However, several clinical studies are being performed to use transarterial embolization as a treatment option for type I endoleaks [26]. Patients require follow-up CT imaging to assess the aneurysm sac size, size of the nidus, diameter of the feeding and draining vessels, and the diameter of the feeding collateral artery to determine if intervention is necessary.

## 3. Liquid Embolics

In general, liquid embolic agents can be classified in two categories: polymerizing liquid embolics and precipitating liquid embolics. In the case of polymerizing liquid embolics, monomer or prepolymer solutions polymerize in the presence of an initiator (ionic solutions, proteins, etc.) into a covalently crosslinked solid material. Polymerizing liquid embolics also include several in situ gelling systems which are thermo-responsive and undergo sol-to-gel phase transition at body temperature. For the precipitating embolics, a solution of preformed polymer in a water-miscible solvent is injected in the body. After injection, the solvent diffuses out and the embolic material precipitates into a solid matrix. Table 1 summarizes the liquid embolics that are discussed in this review.

### 3.1. Polymerizing Liquid Embolics

#### 3.1.1. Cyanoacrylate Glues

The use of cyanoacrylate (CA) glues for polymerization in the presence of blood was proposed in the 1970s [3]. N-butyl-2-cyanoacrylate (NBCA) (Histoacryl, Avacryl and Trufill n-BCA), often referred to as the “Glues”, was introduced as an embolic agent in the 1980s and Trufill n-BCA was approved by the US Food and Drug Administration (FDA) in 2000 for use in the treatment of cerebral arteriovenous malformations [4,27,28] by replacing the carcinogenic Isobutyl-2-cyanoacrylate due to its predictable polymerization qualities, ease of surgical resection, and low toxicity [29,30]. Due to the adhesive nature of NBCA, it can mechanically occupy the intravascular lumen and stop blood flow regardless of blood coagulability [4,31,32].

NBCA monomer is a clear liquid which polymerizes to an adhesive, nonbiodegradable rigid material upon activation by contact with any ionic substances (e.g., blood, saline, ionic contrast media, and vascular endothelium) [4,28] via an anionic or radical mechanism [29,33]. However, in 2003, Levrier et al. showed that anionic and radical polymerization of NBCA for embolization were identical in occlusion rate, but that the radical pathway of polymerization exhibited lower histotoxicity [33]. However, due to the rapid polymerization of NBCA in contact with ionic substances, the therapeutic microcatheter is rinsed with a nonionic solution, such as dextrose solution, to clear it of any blood or contrast medium and prevent the risk of microcatheter entrapment within the artery [2].

NBCA is not radiopaque, so it is opacified by mixing ethiodol, tantalum, or tungsten powder to monitor its flow during injection [2]. In order to control the rate of polymerization, NBCA is mixed with ethiodized oil (Lipiodol or Ethiodol), which can prevent early polymerization within the feeding artery and late polymerization within the draining veins. Since polymerization is too slow when the NCBA concentration is less than 25%, concentrations between 25% and 67% are used depending on the flow characteristics and anatomy of the occlusion target. The rate of polymerization in blood is less than 1 s at 67% NBCA and 6 s at 25% NBCA [4,28]. Alternatively, NBCA penetration into the target area can also be improved by flushing the guiding catheter continuously with dextrose solution during the injection [34,35]. Surgical resection of the vessels embolized with NBCA are simpler as they are easily distinguished from normal vessels and compressible [29,36]. The polymerization of NBCA is exothermic [3,37] and releases formaldehyde, leading to acute and chronic inflammation of the vessel wall and surrounding tissue [2]. Recanalization of NCBA can take place if it is deposited close to the arterial feeder without casting of the whole AVM [2]. NCBA delivery is quite difficult due to its highly adhesive property, very rapid polymerization, and addition of radiopaque agents which interfere with the polymerization [3].

Researchers have worked with NBCA for decades as an embolic agent. In two early studies, reported in 1978 and 1981, NCBA was reported to be successfully used for the occlusion of the renal artery of dog [38,39] and rabbit models [39]. These two studies have also reported the clinical application of NCBA for therapeutic embolization in patients with hypernephroma, achieving permanent occlusion of the renal artery and elimination of hematuria [38,39]. After its acceptance in clinical studies, NCBA has been widely used in cerebral AVM embolization, which has been covered in several review articles [3,4,29]. NBCA has been used as a preoperative embolic agent for the treatment of brain AVMs for a long time. In 1993, after a clinical study on 33 patients, Jafar et al. concluded that the surgical resection of AVM lesions after preoperative NBCA embolization was facilitated due to their higher grade and larger size, thus reducing operative time and intraoperative blood loss without any statistically significant difference in surgical complications or long-term neurological outcome [36]. In a comparative randomized trial study, performed in 1996–1999 on 104 patients and reported in 2002 by the NBCA trial investigators, it was shown that n-BCA is equivalent to PVA as a preoperative embolic agent for the treatment of cerebral AVM [40]. In 2008, Jayaraman et al. reported clinical studies performed from 1995–2005 for the preoperative NBCA embolization of brain AVMs and concluded that embolization of brain AVMs can be performed with a high degree of technical success and low rate of neurologic complication [41]. In 2003, Cantasdemir et al. reported endovascular embolization with NBCA on five patients performed between 1998–2000 for treatment of hematuria in pseudoaneurysms caused by traumatic or iatrogenic injuries of the renal artery branches [42]. The technique was reasonable and effective, as it preserved maximum renal tissue and eliminated the potential risk of nephrectomy.

In 2005, Denys et al. used NBCA for the preoperative embolization of portal vein for partial hepatectomy in patients [43]. Later, in 2017, Miyachi et al. investigated if the success of stereotactic radiosurgery (SRS) is dependent on the performance and quality of preoperative embolization with NBCA. For this, they reviewed SRS performed from 2003–2012 on 73 patients, where it was successful on 43 patients and un-successful on 29 patients and concluded that effective embolization is essential to for successful radiosurgery [44]. In 2007, Razavi et al. successfully used NBCA for the embolization of bronchial arteries to manage hemoptysis [45]. In 2012, Monsignore et al. described a clinical study where they successfully used NBCA as the only embolic agent for arterial embolization of hemorrhagic liver lesions on 8 patients [46]. In 2013, Pietura et al. successfully performed a novel method of endovascular embolization of varicoceles using NBCA glue on 17 patients without any complications, indicating that NBCA embolization of varicoceles is efficient and safe [47]. In another study in 2013, Yata et al. assessed the clinical utility and safety of transcatheter arterial embolization with NBCA for urgent control of acute arterial bleeding in the upper and lower gastrointestinal tract on 37 patients with a high rate of complete hemostasis and a low recurrent bleeding rate [48]. In 2015, Won et al. reported the clinical efficacy of transcatheter embolization of visceral artery pseudoaneurysms using NBCA on 13 patients where all the patients recovered immediately after the process [49]. In a recent clinical study in 2019, Hassan et al. evaluated the efficacy and safety of endovascular embolization of post-tonsillectomy pseudoaneurysm with NBCA glue [50]. In other studies, performed in the same year, the transcatheter embolization of NBCA was successfully used via the pulmonary artery under proximal coil blocking in Rasmussen’s aneurysm [51], and NBCA glue for the transcatheter embolization was used as an alternative therapeutic option in the treatment of esophagomediastinal fistula [52].

#### 3.1.2. Fibrin Glue

Fibrin is a protein polymer formed from the catalytic action of thrombin on fibrinogen [4]. Fibrin glue is a widely used FDA-approved hemostatic agent (Tisseel, Hemaseel), used in surgery, but has also been used as an embolic agent [3]. Fibrin glue, being biodegradable due to its high water content, has only been used clinically as an embolic agent for preoperative embolization [3]. For embolization with fibrin, a double-barreled syringe is used, where one part is loaded with fibrinogen and aprotinin (antifibrinolytic agent) and the other part is loaded with thrombin, calcium chloride, and a visualizing agent [3]. When the contents are emitted during injection, they are mixed in the tip of the syringe and the polymerization begins immediately [3]. The injection rate of the fibrin glue must be optimized. If the injection rate is too fast, it can occlude the catheter, but if it is too slow, it may not solidify within the target vessel and may pass distally into the lungs, liver, spleen, or heart [53]. Typical occlusion time of the fibrin glue ranges from 55 s to 3 min 10 s, depending on the type of the catheter [53]. Due to the biodegradability of fibrin glue, it is not used for AVM embolization as vessel recanalization can be observed over time [3]. The need for double-lumen catheterization during the embolization procedure is another disadvantage of using fibrin glue as the embolic agent [3]. Finally, as fibrin glue is derived from human blood, it carries the risk of blood-borne pathogen transmission and allergic reaction [4,54,55]. Fibrin glue has been used as an embolic agent for the preoperative embolization of the portal vein [56,57], intracranial meningioma [58], bilateral atherosclerotic common iliac aneurysm [59], sinus hemangiomas [60], and sustaining type II endoleaks [61,62,63].

In 1999, Nagino et al. reported a study on 16 patients who underwent preoperative right trisegment portal vein embolization with fibrin glue (Beriplast) for right hepatic trisegmentectomy without any complications [56]. In the same year, Probst et al. successfully used fibrin glue clinically as an embolic agent in a study for the preoperative embolization of intracranial meningiomas in 80 patients [58]. After observing the necrosis of the embolization area via CT scan, the surgery was performed with reduced intraoperative blood loss and operating time. In 2000, Beese et al. reported a successful use of fibrin tissue glue (Beriplast) for endoluminal embolization of bilateral atherosclerotic common iliac aneurysm, without any complications [59]. In this study, Beriplast was introduced endovascularly for treating two large iliac aneurysms in a patient who had a previous abdominal aortic aneurysm repair. In 2002, Kim et al. reported a novel method of removing a large cavernous hemangioma of the cavernous sinus using multiple intratumoral injections of fibrin glue, which was very effective for preventing excessive blood loss during surgery [60]. In 2009, Meyer et al. reported an unconventional closure of a sustaining long-term periprosthetic type II endoleak using fibrin glue after former endovascular repair of an infrarenal aneurysm in one patient [61]. In 2013, Piazza et al. reported a randomized study for evaluating the outcomes of intraoperative aneurysm sac embolization using a standard dose of fibrin glue and coils on 162 patients having risk of type II endoleak during their endovascular aneurysm repair. They mentioned in the report that the process significantly reduced type II endoleak and its complications during short-term and midterm follow-up in patients considered at risk [62]. In 2016, the same group reported another randomized study using a sac-volume-dependent dose of fibrin glue and coils on 126 patients between 2012–2014. In this study, type II endoleak and its complications were significantly reduced during short-term and midterm follow-up in patients [63]. During a clinical study in 2013, Choi et al. successfully used fibrin glue along with vascular occluding coils in one patient for closing a pancreatoduodenal fistula, which remained open after the percutaneous catheter drainage of a pancreatic abscess [64]. They claimed that the procedure is a less invasive, more effective, and better tolerated strategy for high-risk patients. In 2016, Olthof et al. performed reversible portal vein embolization using fibrin glue with different concentrations of the fibrinolysis inhibitor aprotinin in a rabbit model [57]. Portal vein embolization of the cranial liver lobes of the rabbits was performed using fibrin glue+1000, 700, 500, 300, or 150 kunits/mL aprotinin and the results were compared with a permanent embolization using the same experimental set-up. Reversible portal vein embolization in 80% of rabbits was observed in the group of fibrin glue combined with 500 kunits/mL aprotinin. Further, the hypertrophy response was comparable with permanent embolization materials.

#### 3.1.3. In Situ Gelling Type

##### Poly(NIPAAm) Based

Copolymers of poly(N-isopropyl acrylamide) (NIPAAm) are a waterborne in situ gelling polymer system that can be used for endovascular embolization. Although poly(NIPAAm) itself is non-degradable, NIPAAm-based copolymers are ideal as embolic agents as they can be functionalized into degradable [65] and radio opaque [66] polymers. They typically are non-adhesive, non-hazardous, and can reduce recanalization due to swelling inside aneurysms [4]. The gelation technique of NIPAAm-based copolymers can also be designed to take place via a dual mechanism, including physical crosslinking, due to chain entanglement above LCST and chemical crosslinking through a Michael-type addition between olefin groups (e.g., acrylate, vinyl sulfone, etc.). In an example poly(NIPAAm) system and a thiol compound (e.g., cysteamine, QT, etc.), the gelation occurred under physiological conditions [67,68]. Although gels formed by physical crosslinking are usually soft and easy to inject, they are not suitable for embolization treatment due to their tendency to undergo considerable creep in response to the constant stress exerted by blood pressure [69]. On the other hand, chemically crosslinked gels can be difficult to use because they generally require using hydrophilic polymers to inject, and thus may undergo excessive swelling at body temperature in vivo [70]. Therefore, a dual mechanism of the NIPAAm-based copolymers provides a unique platform for endovascular embolization by balancing the injectability at room temperature of hydrophilic materials and the high strength/stability of hydrophobic materials at body temperature. Moreover, the gel strength and gelation time required for endovascular embolization by NIPAAm-based copolymers can be altered by varying the pH, buffer strength, number, and type of thiol and acrylates, concentration of the polymer, and the molecular weights of the polymers [70,71], which make them very promising embolic agents.

In 1996, Matsumaru et al. investigated a NIPAAm-based copolymer, poly(N-isopropylacrylamide-co-N-n-propylacrylamide), as an embolic agent for intravascular neurosurgery [72]. This material has an LCST at 25 °C and so becomes a strong gel at 37 °C due to syneresis. To investigate the occlusion efficacy of the material in vivo, it was delivered in a rabbit kidney through a microcatheter. However, the delivery of the material without gelling inside the catheter was quite challenging. Additionally, the gel volume reduction due to syneresis inside the AVM was another important challenge. In 2000, An et al. developed ultra-high-molecular-weight poly(NIPAAM-co-acrylic acid (AAc)) for endovascular embolization [73]. The polymer, having an LCST closer to body temperature, was expected to be easier to deliver through a catheter. It also showed little or no syneresis at body temperature and pH. However, high water content of the material in the AVM can weaken the gel. Additionally, the solution viscosity of these ultra-high-molecular-weight polymers can prohibit delivery through the microcatheters used in the embolization of neurological AVMs [74]. In 2005, Vernon et al. developed a NIPAAm-based lower-molecular-weight copolymer poly(NIPAAm-co-AAc) for the endovascular embolization of AVMs [74]. The gel strength and shear modulus of the hydrogel (at 37 °C) produced by this polymer can be increased by increasing polymer concentration in solution with a smaller increase in solution viscosity (at 25 °C) compared to increasing the molecular weight of the poly(NIPAAm-co-AAc). This group investigated the relationship of the gel strength and stiffness (at 37 °C) to solution viscosity (at 25 °C) in poly(N-isopropylacrylamide-co-acrylic acid) solutions with regard to acid content, molecular weight, and solution concentration. These gels had sufficient mechanical strength without excessive syneresis and the solution viscosity. Therefore, the mechanical properties of this polymer can be further optimized for delivery into neurological AVMs. In 2005, Li et al. prepared N-isopropylacrylamide (NIPAAm)–N-propylacrylamide (NPAAm)–vinyl pyrrolidone (VP) terpolymers (PNINAVP) of varying ratios as embolic materials for treating AVM [75]. PNINAVP was then combined with the radio-opaque agent iohexol, which produced an integrated bulky hydrogel at body temperature. Satisfactory embolization was observed with 5 wt% 16:16:1H PNINAVP solution containing iohexol in an in vitro embolic model experiment. To evaluate the occlusion of vessels in vivo, this solution was injected through a microcatheter into the rete mirabiles (RM) of six swine. Complete occlusion of the RM was confirmed by angiography immediately after the operation. No recanalization was observed after one month post-operation, and histology data confirmed no acute inflammatory reaction inside the RM and surrounding tissue. All these data suggest the great potential of this polymer in the endovascular treatment of cerebral AVM.

**Figure 1:**
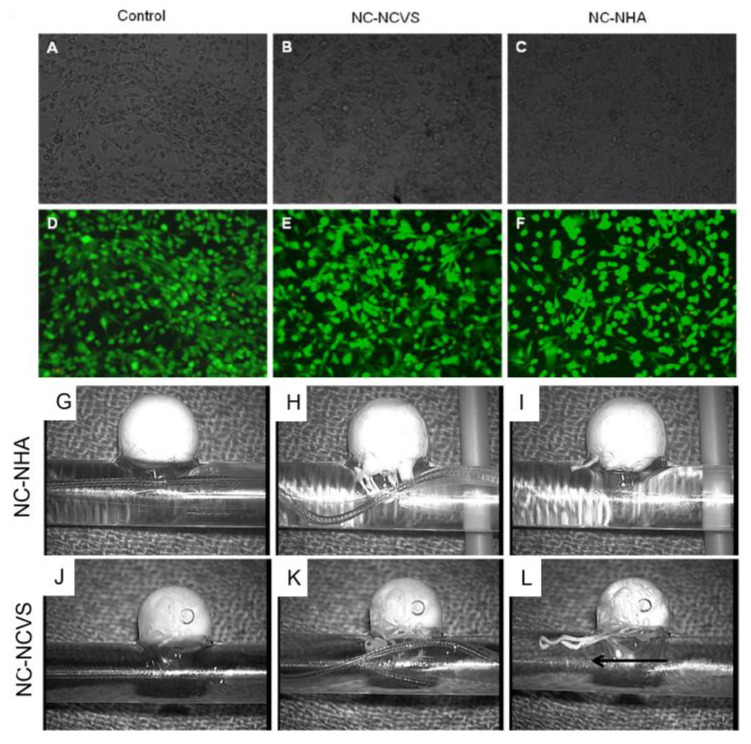
Demonstration of NC-NHA and NC-NCVS gelling systems that undergo physical gelation via thermo-responsive poly(NIPAAm) segment and chemical gelation through an addition reaction between thiols and vinyls. (**A**–**F**) Images of live/dead assay on mouse fibroblasts under (**A**–**C**) bright field and (**D**–**F**) fluorescent for control, NC-NCVS, and NC-NHA systems. (**G**–**L**) Various stages of the in vitro catheter delivery of the NC-NHA and NC-NCVS hydrogel in an aneurysm glass model. (**G**,**J**) Balloon inflated and hydrogel injected in aneurysm sac, (**H**,**K**) balloon taken down, and (**I**,**L**) flow, in direction of arrow, resumed to observe embolization results of the gelling systems.

In 2006, Lee et al. synthesized a NIPAAm-based copolymer poly(NIPAAm-co-HEMA-acrylate) which was mixed stoichiometrically with pentaerythritol tetrakis 3-mercaptopropionate (QT) in 0.1 N PBS solution at pH 7.4 to form a physical–chemical hydrogel [70] via Michael-Type Addition reaction. The resulting thermo-responsive hydrogel had low swelling, improved elastic properties at low frequency, and low cytotoxicity. In 2007, Robb et al. developed another simultaneous gelling system by combining poly(NIPAAm-co-cysteamine) with poly(ethylene glycol) diacrylate (PEGDA) [76]. However, the high pH required for QT solubility for the former system and swelling of the gel due to highly hydrophilic PEGDA for the latter system led to the development of a third simultaneous gelling system [67], developed by Bearat et al. in 2011 [67]. This in situ gelling system consisted of poly(NIPAAm-co-cysteamine) and poly(NIPAAm-co-HEMA-acrylate) that forms a hydrogel when mixed at physiological conditions, mainly at a pH of 7.4 and body temperature of 37 °C. After mixing, the co-polymers undergo physical gelation instantly after exposure to body temperature and then are cured with chemical cross-linking through a Michael-type addition reaction between the thiol group on the cysteamine and the vinyl group on the acrylate. This gelling system, having improved features such as higher mechanical strength, and reduced creep, tunable gel time, and optimized swelling behavior, could potentially be used in endovascular embolization of aneurysms or AVMs (Figure 1).

In 2009, Dai et al. developed thermoresponsive BAB-type HEMA/NIPAAm triblock copolymers (A = NIPAAm, B = HEMA). At higher temperatures, the aqueous solution of BAB copolymers containing shorter PNIPAAm blocks (BAB1-6) failed to form a stable gel. On the other hand, a relatively stable gel was achieved with BAB copolymers, having longer PNIPAAm blocks (BAB1-8) [77]. Furthermore, the polymer showed better gelling capacity in the presence of a radiopaque agent (RA). In an in vivo study, effective occlusion of the cerebral RMs and renal arteries of pigs were observed with the mixture at 5 wt% BAB1-8 and RA. No recanalization was observed for three months after the surgery, and the embolized kidney significantly reduced in volume. A histological assay of embolized kidney demonstrated interstitial fibrosis and calcification as well as the thickening of renal small artery. As a result of these encouraging data, the authors claimed that this copolymer has great potential to be used as an embolic agent for treating AVMs and renal disease.

In 2011, Zhao et al. explored a NIPAAm-based copolymer p(N-isopropylacrylamide-co-butyl methylacrylate) (PIB) nanogel, mixed with iohexol (PIB-I-6150), as a novel blood-vessel-embolic material in the transcatheter arterial embolization (TAE) therapy of liver tumors. Their performance was compared with those of Lipiodol and PVA particles (Ivalon) [78]. The in vivo embolic effect of PIB-I-6150 was evaluated in the renal artery of a rabbit model, showing successful embolization of the peripheral blood vessels. Radiological data indicate that PIB-I-6150 showed better results in peripheral and permanent embolization than Ivalon. TAE therapy with PIB-I-6150 was investigated in the liver arteries of VX2 tumor-bearing rabbits and evaluated by comparison with Lipiodol. Angiographical and histological studies on the embolization indicated the complete occlusion of all levels of blood vessels, including peripheral vessels. Experimental data on tumor volume, necrosis level, and the number of metastatic foci indicated that PIB-I-6150 showed better peripheral embolization than Lipiodol. In 2013, this same group further investigated the distribution, durability of vascular occlusion, and inflammatory reactions of PIB in the renal artery embolization of rabbits [79]. The proper injection rate for the embolization was first investigated on nine rabbits, and then embolization was performed with the established injection rates on twenty rabbits. To evaluate the long-term effect of the embolization, angiography and pathologic examination of the kidneys were performed at one week, and at one, two, and three months after embolization. After three months, the occlusion remained homogeneous and persistent without severe inflammatory changes. No complications, including recanalization, disruption of the vessel wall, or subintimal bleeding, were observed after three months, concluding PIB as a suitable material for intravascular embolization.

In 2012, Bearat et al. reported a comparative study of the properties between a pair of NIPAAm-based simultaneously physically and chemically gelling polymer systems. The synthesized polymer gelling systems includes poly(NIPAAm-co-cysteamine) with poly(NIPAAm-co-HEMA-acrylate) (NC-NHA) and poly(NIPAAm-co-cysteamine) with poly(NIPAAm-cysteamine-co-vinylsulfone) (NC-NCVS). Some differences were noted in terms of the rheological and kinetic studies [80]. The NC–NCVS system had faster gelation kinetics and produced stronger gels; however, no differences were found in the swelling, degradation, or cytotoxic properties. In 2013, the same group published an in vivo study with an NC-NHA gelling system for endovascular embolization of cerebral aneurysms in a swine model [68]. The gel was easily injected via catheters into the aneurysms and the gels remained in the aneurysm after deflation of the balloon and reintroduction of flow. However, full occlusion did not occur, and slight recanalization was observed almost immediately after the procedure.

##### PPODA-QT/PEGDA-QT Based

Vernon et al. developed a novel liquid embolic polymerizing system using poly(propylene glycol) diacrylate (PPODA) and pentaerythritol tetrakis (3-mercaptopropionate) (QT) for the occlusion of AVMs [81]. Both PPODA and QT monomers are liquid at room temperature, but upon mixing in the aqueous solution of higher pH, a chemical reaction occurs via Michael-type addition between these two multifunctional compounds to form a hydrogel matrix. At higher pH, the hydroxyl group deprotonates the thiol group of QT to form a nucleophile which then reacts with the acrylate group of PPODA via Michael-type addition reaction [82]. Reaction kinetics and the rate of gelation can be controlled by changing the pH of the aqueous solution.

**Figure 2:**
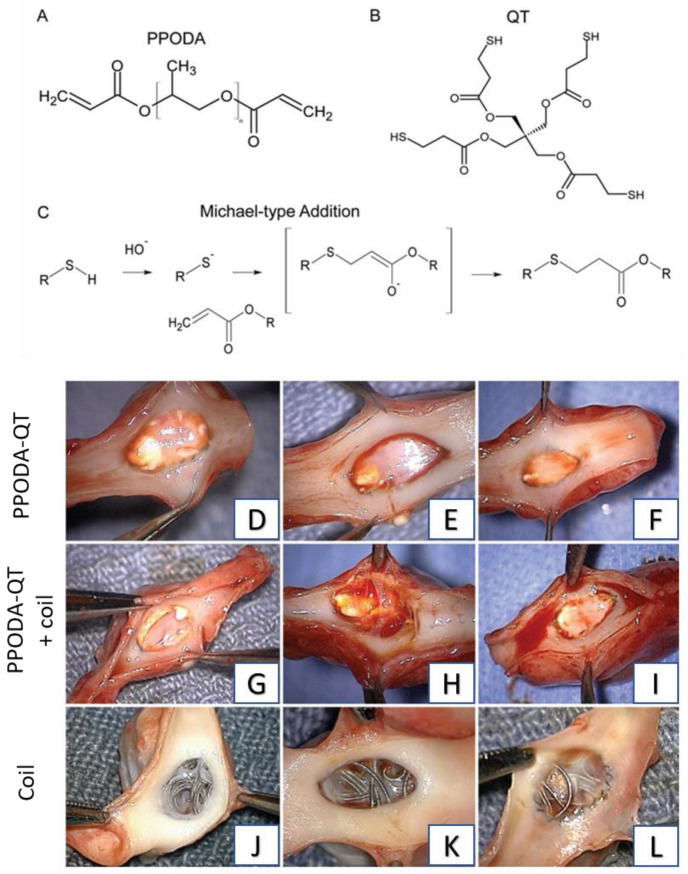
Components (**A**,**B**) and reaction scheme (**C**) of PPODA−QT. Photographs of explanted aneurysm samples. The PPODA−QT aneurysm samples (**D**–**F**) all show a smooth surface in the ostium. The coil + PPODA−QT aneurysms (**G**–**I**) show excess PPODA−QT protruding into the parent vessel in two samples, resulting in rough surfaces (**G**,**H**), while one sample displays no PPODA−QT protrusion and a smooth surface (**I**). The coil-only aneurysms (**J**–**L**) show the least neointimal tissue overgrowth. Reprinted with permission from Ref. [86]. 2013, J. Neurosurgery.

In 2010, Riley et al. investigated this PPODA-QT-based in situ gelling system formulated with injectable contrast agents for intracranial aneurysm embolization [83]. For this purpose, two commercially available contrast agents, Conray and Omnipaque, were pH-adjusted to basic conditions, pH 10.8 and pH 12.2, respectively, and used as initiating solutions with the PPODA-QT system. Omnipaque-formulated gels require a higher pH for the system to solidify in comparison to the Conray-formulated gels in the same time frame due to its lower buffer capacity. Later in 2012, Brennecka et al. investigated the feasibility of delivery in a mock aneurysm, the cytotoxicity, the swelling, and the degradation behavior of these two gelling systems [84]. Although both the PPODA–QT systems were easily deliverable to mock aneurysms, cytotoxicity results indicated that Conray-formulated gels are initially less toxic than Omnipaque-formulated gels but show greater long-term swelling and faster degradation over time. As initial cytotoxicity plays a vital role in successful in vivo treatment, Conray-formulated gels may be better for in vivo neointimal tissue growth. In the same year, this group used the Conray-formulated PPODA-QT gelling system for in vivo aneurysm embolization in a swine model for initial biocompatibility and delivery strategy analysis [85]. This was the first in vivo use of a PPODA-QT gelling system as an embolic agent for aneurysm treatment. Three delivery strategies were used to embolize aneurysm of the swine model, including complete filling of the aneurysm, sub-completely filling (80–90%), and three-dimensional coil placement, followed by polymer embolization with PPODA-QT gelling system. After embolization the swine were followed for 1 month. PPODA-QT showed excellent biocompatibility and among these three strategies, sub-completely filling aneurysms to 80% to 90% capacity proved to be a safe and effective delivery strategy. These successful results show this gelling system to be a potential viable alternative to current embolic materials. In 2013, to investigate the long-term efficacy and evaluate the effectiveness of PPODA-QT on a more clinically appropriate animal model, the same group used the same Conray-formulated PPODA-QT gelling system for in vivo embolization of lateral wall aneurysms in canines in a 6-month study [86]. For this purpose, surgically created experimental aneurysms were embolized with only PPODA-QT (3 dogs), with PPODA-QT after placing a 3D framing platinum coil (3 dogs), and with traditional platinum coils (3 dogs). Although all aneurysms showed 100% occlusion immediately as well as at 6 months through angiography, the coil + PPODA-QT group indicated polymer protrusion into the parent artery. Additionally, the PPODA-QT group produced smooth ostial surfaces, facilitating more complete neointimal tissue coverage over the aneurysm necks, while the latter two groups showed rough and discontinuous ostial surfaces, hindering neointimal tissue coverage. Finally, according to the gross observation and histological data, embolization with PPODA-QT exhibited better recanalization than traditional endovascular coiling (Figure 2).

In a study performed in 2013, Soodak et al. developed a poly(ethylene glycol) diacrylate (PEGDA)-QT gelling system using PEGDA of different molecular weights (PEGDA575 and PEGDA700), and investigated their characteristics in [87]. Data indicated that PEGDA700-QT showed faster degradation and swelled quicker than PEGDA575-QT gels, suggesting that PEGDA575-QT would be a safer material for aneurysm embolization. However, PEGDA700-QT exhibited a steady protein release profile as well as better cytocompatibility than PEGDA575-QT, making it more promising for embolization. Therefore, continued study of both systems in vivo is warranted to gain a better understanding and more conclusive outcomes.

#### 3.1.4. Shear-Thinning Biomaterials

Shear-thinning biomaterials were developed through collaborations with the Mayo Clinic, Harvard-MIT [88]. These nanocomposite hydrogels contain a mixture of gelatin and silicon nanoplatelets to promote embolization. This is an injectable material that does not rely on thromboembolization as a means of occlusion. These materials may be competitive with metallic coiling, as these materials have been shown to withstand fragmenting when at hemodynamic pressures [88]. The hydrogels undergo electrostatic interactions between the gel components and readily flow through injection shear stresses and forms a spontaneous hydrogel that conforms to the surrounding anatomy leading to embolization [89].

#### 3.1.5. Silk Elastin Protein Liquid Embolic (SELP)

Theratarget produces a silk elastin hydrogel with low viscosity. This polymer system is designed to treat hypervascular tumors that are unresponsive to systematic chemotherapy. The polymeric treatment is delivered in a transcatheter arterial chemoembolization (TACE) procedure. This silk-elastin-like protein polymer (SELP) is part of an injectable liquid-to-solid transformation used for the treatment of hypervascular tumors. The system provides localized delivery of chemotherapeutics to deep penetration and occlusion of the surrounding feeding vasculature. The low-viscosity chemoembolic system is also compatible with many smaller-gage catheters for access to targeted vessels [90]. Silk elastin like proteins belong to the family of genetically engineered recombinant protein polymers that are tunable in their mechanical properties [91,92]. These biomaterials consist of repeating silk-like and elastin-like peptide blocks. The physiochemical properties can be tuned by varying the silk–elastin ratio and molecular weight to incorporate drug delivery. Recombinant proteins are also biodegradable and can be tuned to respond to environmental stimuli such as temperature, pH, ionic strength, redox, enzymatic stimuli, and electrical fields. Lastly, SELP materials are of interest because they can form hydrogels at physiological conditions and be processed into a variety of material formats, such as nanoparticles, thin films, and scaffolding [91].

#### 3.1.6. Instylla Embrace HES

The Instylla Hydrogel System is used for the embolization of tumors and organs with a hydrophilic hydrogel derived from functionalized glycol capable of rapid formation [92,93,94]. Unlike Onyx, which relies on precipitation for occlusion, the delivery of this self-reacting hydrogel can be paused, offering greater procedural control. The hydrogel is soft and mimics the feel of the surrounding tissue, thereby not contributing to a thrombolytic response. Furthermore, animal studies have shown embolization of vasculature below ten microns in size. Microvention has also been working on a next-generation hydrocoil system. This system consists of a platinum core with hydrogel integrated to enhance packing density and healing within the aneurysmal sac. Randomized and controlled clinical trials (600 patients) are currently underway (Hydrogel Endovascular Aneurysm Treatment Trial, HEAT) comparing the hydrocoil to a bare platinum coil for subjects with ruptured and unruptured 3–14 mm intracranial aneurysms that are amenable to coiling [94]. Embrace HES is an investigational device intended to be used to embolize hypervascular tumors in vessels ≤ 5 mm [94]. Embrace HES consists of two low viscosity liquid precursors that solidify when simultaneously delivered into blood vessels, forming a soft hydrogel that fills the vessel lumens during the embolization procedure. The Embrace HES embolization uses no solvents, does not need sizing to the vessel diameter, and eliminates the possibility of catheter entrapment. Its main components are water and polyethylene glycol (PEG) [95,96].

#### 3.1.7. Alginate

An initial report investigating the use of calcium alginate (CA) as an endovascular liquid embolic was provided by Becker et al. in 2001 [172]. By 2002, this group reported a study of alginate flow properties to further support the use of alginate for this application [172]. This report concluded that due to shear thinning effects, alginate could be formulated to be deliverable through typical microcatheters used for endovascular embolization, thus motivating continued study for this application [173]. That same year, Becker et al. assessed the stability of calcium alginate in a swine rete mirabile (RM) model to mock AVMs. The injectability, radiographic visualization, mechanical stability, and short-term biocompatibility of the calcium alginate were studied in the RM [174]. The material could be injected into the RM in controlled fashion, leaving the contralateral portion of the RM and the circle of Willis open [174] with one week survival results demonstrating mechanical stability [174].

Around the same time, other groups were also investigating alginate for endovascular embolization. In 2003, Raymond et al. explored the potential use of alginate for endovascular treatment of aneurysm while providing simultaneous drug delivery of platelet-derived growth factor-BB (PDGF-BB) or transforming growth factor-β1 (TGF-β1) [97]. In this study, angiographic results and neointima formation at 3 weeks were compared after intraoperative embolization of canine lateral wall aneurysms with alginate blocks between controls without drug, PDGF-BB, and TGF-β1. Although this study showed the ability to deliver the drugs in vitro and in vivo, stability of the material was insufficient for aneurysm embolization which led to carotid emboli. Thus, the authors concluded that further development was needed before use in intracranial aneurysms [97].

By 2005, Becker et al. reported a longer-term swine RM model [98]. In 8 of 9 animals, angiography and histology showed complete occlusion of the RM feeding vessel up to 6 months post-embolization. Though minor bioactive response was seen at 1 month, the study showed no degenerative or inflammatory responses at that time. Further, at 6 months, there was moderate fibrous tissue formation around the alginate that provided further sealing off of flow to the embolized areas of the RM [98]. This study provided further justification to pursue CA for AVM embolization.

In 2007, Becker et al. conducted a preliminary study to use CA as an embolic agent in a swine side-wall aneurysm model [99]. In 10 animals, angiography and histology showed complete aneurysm embolization in 8 animals and 50% filling in the other 2. Angiography and histology showed complete occlusion and neck healing at 90 days for 8 animals [99]. However, the delivery of the CA required a complex 2 lumen catheter with one lumen injecting the alginate and the other calcium chloride. The timing of the calcium chloride delivery had to begin slightly ahead of the alginate injection. Gelation of the alginate was rapid and did not allow for additional material to be injected that would adhere well to a volume of CA already delivered. Thus, a seam remained between the gel volumes that did not allow optimal filling of the aneurysm and prevention of blood flow into the aneurysmal sac. Such a situation could and did produce emboli in several animals. This composition of CA was tried in one patient, but its radio-opacity and visualization were not optimal and thus its further use was abandoned.

In a more recent study in 2020, Fan et al. investigated an injectable and radiopaque liquid metal (Gallium (Ga) and Indium (In))/calcium alginate hydrogel for endovascular embolization and tumor embolotherapy [100]. LM droplets prepared were comprised of 75.5% Ga and 24.5% In. The LM droplets were combined with CA solutions to prepare a LM/CA mixture. Results in the back of 12-week-old BALB-C mice showed the LM/CA was safe and had low toxicity during a 4-week study [100]. An additional blood coagulation study and hemolysis test suggested that the LM/CA was also hemocompatible [100]. Use of the LM with the CA provided radiopacity of the material confirmed with microCT and X-ray imaging. The effect of embolization using this material was observed using auricular veins in rabbit ears. Blood flow in the vessel was eliminated and there was no visible injury to the vessels. After 14 days, there was no obvious inflammation or infection observed related to the embolization [100]. In a tumor-bearing rabbit model, embolization of the tumors with this LM/CA dramatically inhibited tumor growth and the tumors were eliminated after 2 weeks [100].

### 3.2. Precipitating Liquid Embolic Agents

#### 3.2.1. Eudragit-E

Eudragit^®^ (marketed by Evonik) has been used as a coating material for orally administered tablets since 1954 [101], which has been approved by the United States Food and Drug Administration in 1968 [102,103]. Eudragit-E (Eudragit-E 100; Evonik, Essen, Germany) is a nonadhesive liquid embolic material, originally developed by Yamashita et al. in 1996, which is a cationic copolymer of dimethylaminoethyl methacrylate, butyl methacrylate, and methyl methacrylate with a ratio of 2:1:1 that is dissolved in 50% ethanol and 50% iopamidol [104]. Inside the body, the ethanol diffuses out of the solution and Eudragit-E precipitates rapidly forming a soft elastic sponge within seconds. Yamashita et al., in their research, demonstrated the successful transcatheter embolization of 4 canine and 52 rat renal arteries without any recanalization and acute inflammatory reaction within 1 week, but mild to moderate reactions in the subacute and chronic stages [104]. In 1998, Satado et al. used Eudragit-E for the embolization of renal arteries of a rabbit model, and also investigated the effect of ethanol on the body post-embolization process [105]. For this purpose, they injected Eudragit-E mixed with 50% ethanol + 50% iopamidol in the first group, only 50% ethanol + 50% iopamidol mixture in a second group, and 50% saline + 50% iopamidol mixture in a third group. It was observed that the arteries of the rabbits in the first group were occluded successfully without recanalization. However, acute vasculitis was observed in both the first and second group receiving the ethanol/iopamidol mixture, but not in the third group with the saline/iopamidol mixture. Additionally, the second group also showed small infractions in the kidney of the rabbits. The researchers demonstrated that despite the drawbacks of the ethanol, it can potentially enhance the occlusive effects of Eudragit-E if it is properly injected. In 2007, Arakawa et al. reported endovascular embolization of swine retia mirabilia (RMs) from the injection of Eudragit-E [106]. After embolization, RMs were evaluated both angiographically and histopathologically acutely (6–24 h) at 30 days and at 90 days after embolization. Although, no retention or adhesion of the microcatheter was observed during the procedure, inflammation, and endothelial damage were observed in all the samples. Partial recanalization was observed in the 30-day group but not in the 90-day group. Arterial fibrosis, calcification, and disruption of internal elastic lamina were also observed in the 30- and 90-day specimens. Despite all these complications, there was no evidence of Eudragit-E extravasation or hemorrhage, and therefore the authors warranted Eudragit as a potentially useful embolic material for brain AVMs. In 2015, Tamura et al. reported endovascular embolization of human brain AVMs with Eudragit-E, which was performed on twenty-two patients’ brains from 1998–2014 [102]. During the endovascular procedure, vasospasm was rarely observed and no catheter trapping on the cast occurred due to the non-adhesive nature of Eudragit-E. Therefore, the injection was able to stop and restart at any time without pulling off the microcatheter. In their process with 22 patients, the complete obliteration rate with endovascular treatment alone was 27.3%, whereas it was 72.7% with/without additional treatments, including the use of coils, gamma knife radiosurgery, and planned surgical excision. The overall complication rate in this study was 31.8% and mortality rate was 4.5%. The rate of complications directly related to the embolization process was 10.0% (3/30).

Although Eudragit-E has been used for endovascular embolization of brain AVMs for some time, it is currently not widely used because of safety and feasibility. The 10% Eudragit-E mixture is not supplied in ready-to-use vials and its use needs to be approved by an Institutional Review Board as well as prior informed consent from patients and their family [102]. According to Arakawa et al., the inflammatory responses are mainly due to the Eudragit polymer, which might cause the interaction between positively charged polymers and negatively charged blood cells [106]. They have also proposed that the ethanol/iopamidol mixture did not cause any endothelial damage and was not responsible for the inflammation during/after the process [106].

#### 3.2.2. Squid

Squid and Squid Peri (Emboflu, Gland, Vaud, Switzerland) are non-adhesive, precipitating liquid embolic agents composed of ethylene vinyl alcohol copolymer (EVOH) in dimethyl sulfoxide (DMSO) solvent with suspended micronized Tantalum powder for radiopacity [107]. Squid and Squid Peri is used for neuro applications and peripheral use, respectively [82]. Due to the use of micronized tantalum powder, the sedimentation rate is slower than the regular tantalum powder, resulting in a homogenous mixture that can be retained for a longer time and hence provide longer working time [107]. Squid is available in four formulations, including Squid 18, Squid 12, Squid 18LD, and Squid 12LD [82,107]. Squid18 and Squid 12 have viscosities of 12 mPa s and 18 mPa s, respectively [82]. Squid 18LD and Squid 12LD have the same viscosities as Squid 18 and Squid 12, respectively, but contain 30% less tantalum powder [107]. Squid 18 is used for standard AVM embolization [107] or treatment of endoleaks [82], whereas Squid 12, being less viscous, can penetrate deeper to reach distal micro vessels and can be injected through small feeders [107]. Squid 18LD and Squid 12LD allow easier visualization as they reduce the over-saturation of tantalum powder [107]. All four Squid formulations show reduced beam hardening artifacts on post-embolization CT scans compared with Onyx, which was further investigated and confirmed by a comparative study in a controlled in vitro model [108]. Despite the improved properties over the well-established Onyx treatment, Squid has not found widespread use in a clinical setting, likely due to its high cost and requirement of DMSO-compatible microcatheters [82].

Squid has been used as a liquid embolic agent for AVM, AV fistulas, and tumors. In 2014, Akmangit et al. reported a study with 28 patients with AVMs and AV fistula, who were treated with Squid 18 and Squid 12 [109]. No technical problems were observed during the procedure. Although there was no mortality, the total obliteration rate of the AVMs was 37.5% and of fistulas was 66%. Overall, Squid was reported to be a safe and effective embolic agent for the treatment of cerebral AVMs and AV fistulas with satisfactory obliteration rate. In 2017, Kannath et al. reported the use of Squid as a liquid embolic for the percutaneous embolization of scalp arteriovenous malformations [110], which are traditionally treated by surgical approaches. Complete obliteration of scalp AVM was possible due to the non-adhesive nature of Squid and the formed cast was aesthetically appealing to the patient. The authors suggested that this treatment process could be considered as the primary treatment option for patients with scalp AVM who are unwilling for surgery. In the same year, Torcia et al. reported their first experience with Squidperi for the endovascular embolization of spontaneous iliopsoas hematoma in one patient [111]. After the embolization of the bleeding vessels, complete exclusion of the diseased vessels was achieved without any complications after the procedure. In 2019, Venturini et al. reported the use of Squid for the transcatheter embolization for several abdominal diseases in 30 patients [112]. For this purpose, Squid 18 or Squid 12 were used either combined with other embolic agents such as poly vinyl alcohol (PVA) particles, coils, and amplatzer plugs or alone, for AVMs, portal vein embolizations (PVEs), visceral artery aneurysms (VAAs), type 2 endoleaks, preoperative embolizations, acute arterial bleeding, and female varicocele. Although, over 30 days, successful embolization was observed with low complication rate, future studies with a larger group of patients are necessary to establish Squid as an embolic agent in each specific abdominal disease. In 2020, the same group has reported the embolization of splenic artery aneurysms (SAA) with Squid plus detachable coils in a separate study with 12 patients [113]. Transfemoral embolization was performed with a microcatheter without any major complications or fatal events. For all the patients, complete exclusion of the aneurysmal segment was observed after the process and no aneurysm revascularization was observed at 1 month and 6 months, confirming the effectiveness of Squid plus coils for SAA embolization. In another study performed in the same year, this group reported the embolization of two coexisting intraparenchymal renal artery aneurysms with Squid and coils [114]. In 2020, Lozupone et al. reported endovascular treatment of cerebral dural arteriovenous fistulas (cDAVs) using Squid 12 in 19 patients without any major periprocedural complications [115]. Among these 19 patients, mid-term follow-up was performed in 15 patients. Although complete occlusion of the cDAVFs was confirmed in 13 patients, a small relapse was detected in 2 of the initially cured patients. The results indicated that the endovascular treatment of the cDAVFs using Squid 12 was effective and safe with a high rate of complete occlusion of the cDAVFs. Although Squid has been used successfully for endovascular embolization, a few incidents of complications were also reported. In 2019, Jabre et al. reported the first case of a complication when treating brain AVM with Squid [116]. A rare and serious complication of brain abscesses was found around the embolized brain AVM. This complication was treated with complete safe removal of the embolic material and antibiotherapy. In 2021, Moreno-Paredes reported the first clinical case of permanent facial paralysis secondary to embolization with Squid 12 [117].

#### 3.2.3. Onyx

Onyx (Medtronic Inc.) is a poly(ethylene-co-vinyl alcohol) (EVA) copolymer-based liquid embolic system which is dissolved in DMSO [4,82]. Onyx contains suspended micronized tantalum powder for radiopacity which must be agitated for a minimum of 20 min to obtain a homogeneous suspension prior to delivery [82]. Upon delivery, the polymer precipitates out of the solution, resulting in a gel with a sponge-like consistency, due to the gradient diffusion of DMSO in the bloodstream with the entrapment of the suspended tantalum powder [4,82]. This sponge-like gel is ideal for use in occluding soft vessels and the entrapped tantalum powder provides long-term radiopacity, making it easily located by X-ray imaging for further procedures [82]. Onyx is considered to provide permanent occlusion that can be stable for up to 5.25 years [118]. In a recent review, the advantages of Onyx in endovascular bleeding management have been described [119].

Onyx is marketed in different formulations, including Onyx 18, Onyx 34, and Onyx 500, with viscosities of 18 mPa s, 34 mPa s, and 500 mPa s, respectively [82]. Low-viscosity Onyx can penetrate a long distance, deep in the nidus [120]. On the other hand, high-viscosity Onyx has the potential to resist recanalization of the material [121]. Therefore, physicians use these formulations for the appropriate applications based on their properties. As examples, low-viscosity Onyx 18 is used for treatment requiring a high degree of distal penetration and high-viscosity Onyx 500 is used for the embolization of wide-neck aneurysms [82]. Although Onyx has a lower inflammatory response than NBCA and its non-adhesive nature prevents the blockage of microcatheters, the use of DMSO as the carrier solvent makes its use challenging due to causing vasospasms [82,122,123]. However, vasospasms can be can be prevented by using the recommended slow injection rate [124] of 0.16 mL min^−1^ [82]. Additionally, the injection of Onyx requires DMSO-compatible specialized syringes and microcatheter to avoid the decomposition caused by the solvent [82,123].

In 1990, Taki et al. developed an EVA-based embolic agent which was clinically applied in three cases of AVM embolization successfully [16]. Since then, Onyx^®^ has been used successfully for the treatment of cerebral AVMs [125,126,127,128], peripheral AVMs [129,130,131,132], AVFs [11,12,17,133,134], intracranial aneurysms [22,24,135,136], renal artery aneurysm [1,140], bleeding from ruptured aneurysms [138,139], endoleaks [25,140,141,142,143,144,145,146,147,148,149,150], and preoperative embolization [18,19,25,151,152,153].

Onyx has been widely used in the treatment of cerebral AVMs. In 2011, Xu et al. reported their experiences in the treatment of brain AVMs with Onyx embolization on 86 patients [125]. They concluded that Onyx has moderate obliteration rates (18.6%), but it might be more effective as a presurgical embolic agent for selected large AVMs. In 2013, Soltanolkotabi et al. reported Onyx embolization of intracranial AVMs in 25 pediatric patients and suggested that Onyx embolization can be performed safely with a low rate of permanent morbidity [126]. In 2017, Aoude et al. reported the treatment of brain AVMs with Onyx for 53 patients, where they found the results to be satisfactory with a complete obliteration rate of 20.08% [127]. In 2017, Singfer et al. described primary Onyx embolization (with or without radiosurgery) in 61 patients with unruptured brain AVMs with a high occlusion rate [128].

In 2004, Numan et al. reported endovascular transcatheter embolization of congenital peripheral VMs in nine patients, concluding that Onyx is a promising embolic agent and advantageous over conventional embolic agents [129]. In 2017, Kilani et al. reported endovascular embolization of high-flow peripheral AVMs with Onyx in 19 patients, of which complete devascularization was observed in 12 patients [130]. In 2018, Ne et al. studied 50 patients and reported that Onyx is feasible and safe in the peripheral arterial or venous vasculature for both hemostatic and non-hemostatic sites [131]. In 2019, Giurazza et al. reported their 10-year experience of transarterial embolization of peripheral high-flow AVM with Onyx in 16 patients and concluded Onyx to be safe and technically effective for the process [132].

In 2010, Wang et al. reported the endovascular embolization of congenital brain arteriovenous fistulas (BAVF) with a combination of Onyx and detachable coils/balloon-assisted coils in 15 patients [11]. After the process, angiographic obliteration was immediately observed in all patients and the clinical follow-up indicated no BAVF-related complications. No new detectable neurological deficits indicated that the process was feasible, safe, and effective. In 2011, Dalyai et al. reported the first novel and successful treatment of scalp AVF using a balloon-protected transvenous embolization with Onyx and concluded that the technique might be useful when transarterial embolization, direct puncture, or surgical excision are not practical or possible [12]. In 2013, Kim et al. reported Onyx embolization of dural AVF in association with coils in one patient, resulting in complete occlusion of the AVF [133]. In 2018, Johnson et al. summarized several case studies regarding the embolization of cranial dural arteriovenous fistulas in 96 patients with different embolic agents, including 65 patients with only Onyx, exhibiting the overall complete obliteration rate of 89.6% with a residual fistula rate of 2% [17]. In the same year, Kawamura et al. published a case report of the transarterial embolization of dural AVF of sinus confluence in a safe and effective way [134].

In 2005, Weber et al. reported the treatment of 22 unruptured wide-necked intracranial aneurysms with Onyx-500, resulting in a 91% occlusion rate without permanent severe morbidity and mortality [22]. In 2008, Eddleman et al. reported two pediatric cases with infectious intracranial aneurysms that were embolized successfully and safely with either Onyx alone or in combination with platinum coils [24]. In 2015, Patel et al. reported the treatment of a large traumatic intracranial pseudoaneurysm with Onyx-500 [135]. Using this treatment, immediate occlusion of the aneurysm was achieved, and the parent artery was spared from sacrifice. In 2019, Lu et al. reported the successful embolization of a traumatic pseudoaneurysm of the middle meningeal artery using Onyx in one patient [136].

In 2003, Lupattelli et al. reported the successful Onyx embolization of a renal artery aneurysm, where complete exclusion was achieved immediately without any distal embolization or intra-aneurysmal flow [137]. In 2007, Rautio et al. performed transcatheter embolization with Onyx successfully to treat a clinically silent renal artery aneurysm and observed complete occlusion of the aneurysm at five-month follow-up [1].

In 2010, Jaratua et al. reported Onyx embolization in a patient for the treatment of ruptured splenic artery aneurysm where complete embolization of the aneurysm and the stoppage of the active bleeding was observed [138]. In the same year, Larzon et al. reported internal sealing of ruptured aortic aneurysm with Onyx in two patients, resulting in the occlusion of bleeding [139].

Onyx has been used as a monotherapy for the treatment of type II endoleaks since 2001 [140]. In 2001, Martin et al. published their experience of treating six endoleaks with Onyx where complete endoleak occlusion was achieved in five cases [141]. In 2007, Ling et al. reported endovascular embolization with Onyx to obliterate a large type II endoleak [142]. In 2014, Khaja et al. reported the use of Onyx embolization for the treatment of type II endoleak after endovascular repair of the thoracic and abdominal aorta with 88.9% technical and clinical success [143]. Recently, in 2021, Scallan et al. published a review of prospectively collected data from a vascular surgery database regarding the comparison between endovascular embolization with Onyx vs. coils to treat type II endoleaks and concluded that Onyx-embolized sites are less prone to further reinterventions than with coil embolization [144]. Again in 2021, in a comparative study, Alobaidi et al. described the superiority of intrasac Onyx embolization in comparison with the other embolization agents and techniques [145].

Onyx has been widely used for the treatment of type II endoleaks, but there are a few reports of its use in type I endoleaks. In 2010, Grisafi et al. reported a successful Onyx embolization of a type I endoleak in one patient who declined the option for surgical repair [146]. In 2011, Henrikson et al. performed Onyx embolization to treat type I endoleaks during endovascular aortic repair in six patients [147]. Although the initial experience of the study was promising, longer follow-up with more patients was necessary for further evaluation. In 2014, Cobb et al. presented the successful treatment of a large inoperable type I endoleak in the presence of a mycotic aneurysm sac using Onyx as the embolic agent without any complications [148]. In the same year, Eberhardt et al. reported successful transcatheter embolization of type I endoleaks using Onyx in eight patients [149]. In 2017, Ameli-Renani et al. reported transcatheter embolization of type I endoleaks in 25 patients after endovascular aortic repair [150]. Although, in all cases, immediate technical success with complete isolation of the endoleak was achieved, some complications were observed during follow-up. Overall, the authors claimed this technique to be a safe, feasible, and sustainable treatment option for patients who are unsuitable for standard methods of type I endoleaks treatment. In 2018, the same group published a report presenting the available case reports in the literature for the treatment of type I endoleaks using different embolic agents and concluded that, although the technical and clinical success rates were high, the durability was questionable due to lack of long-term follow-up [25].

Onyx has been widely used for preoperative endovascular embolization. In 2008, Gore et al. published a review of preoperative embolization of cranial and spinal tumors using Onyx, performed over a 19-month period on 10 patients, and concluded Onyx to be advantageous due to its deep penetration ability, causing extensive tumor infarction, embolizing extensive portions of the tumors, and the safety of catheter withdrawal after the process [151]. In 2012, Rangel-Castilla et al. demonstrated the preoperative Onyx embolization of head, neck, and spinal tumors in 100 patients, in an effective and safe way, by a transarterial route or direct puncture [152]. All embolizations were performed in a single session without any mortality or major complications. In 2013, Clarençon et al. reported their initial experience in the treatment of spinal metastases close to the anterior spinal artery (ASA) in two patients [19]. Onyx-18 was injected into hypervascular posterior arch spinal metastases near ASA by direct puncture, resulting in satisfactory devascularization of the lesions, and within 48 h the metastases were surgically removed in both cases without any complications. In 2016, Fusco et al. reviewed their experience with pre-surgical Onyx embolization of extra-axial tumors in five patients where extensive devascularization was achieved and confirmed the safety and efficacy of Onyx in preoperative tumor embolization [18]. In 2018, Luzzi et al. demonstrated the preoperative embolization of 27 grade III Spetzler–Martin brain AVMs using Onyx [153]. Preoperative Onyx embolization facilitated the surgical management, with mean embolization-related obliteration rate, morbidity, and surgical obliteration rate being 28.8% 3.7%, and 92.6%, respectively. However, in 2021, Jen-Chen et al. performed an extensive study to compare the outcomes of the embolization of brain AVMs, with Onyx vs. without Onyx, in pre-stereotactic radiosurgery and concluded that the impact of Onyx embolization was not different compared with non-Onyx embolics [154].

In some cases, Onyx embolization was performed for the management of hemoptysis. In 2010, Khalil et al. shared their preliminary experience of systemic arterial embolization with Onyx in 15 patients with hemoptysis, where it was controlled in 14 cases [155]. In 2021, Mattay et al. described successful Onyx embolization of the bronchial artery in a patient with massive hemoptysis without reflux [156].

Several comparative clinical studies between Onyx and NBCA were reported to investigate the superior efficacy of these materials for embolization. In 2010, Loh et al. reported a randomized trial of Onyx and NBCA for embolization of cerebral AVMs in 117 patients [157]. In total, 54 patients were treated with Onyx and 63 patients were treated with NBCA for presurgical endovascular embolization. More than 50% AVM volume reduction was observed in 96% with Onyx and 85% with NBCA, whereas the secondary end points of resection time and blood loss were similar, concluding that Onyx is equivalent to NBCA in safety and efficacy as a preoperative embolic agent. However, another investigative study performed by Natarajan et al. on 32 patients raised questions about the permanence of occlusion after Onyx embolization due to the higher rate of recanalization and the presence of chronic foreign-body giant cells in comparison to NBCA [158].

#### 3.2.4. PHIL^TM^

PHIL™ (Microvention, Tustin, CA, USA) is a non-adhesive liquid embolic agent comprised of a biocompatible copolymer, poly(lactide-co-glycolide), and poly(hydroxyethyl methacrylate) (PHEMA), covalently bonded to an iodine component for radiopacity and dissolved in DMSO [10,159]. It was introduced in 2015 and is available at concentrations of 25, 30, and 35 wt%, with viscosities of 16, 36, and 72 cSt and embolic capacities of 0.85, 0.87, and 0.94 mL, respectively. All compositions come in a pre-filled sterile syringe containing 1 mL of PHIL™ system that does not require prior preparation or shaking [10,93]. The iodine radiopaque agent, triiodophenol, is covalently bonded to PHIL™. Thus, there is no risk of radiopaque agent aggregation or precipitation regardless of time of usage, resulting in a more homogenous fluoroscopic appearance as compared to Onyx™ [160]. Therefore, PHIL allows more consistent visibility than Onyx [93,160]. Iodine is also more advantageous compared to tantalum during postinterventional imaging because it produces significantly fewer artifacts in CT scans and no artifacts in GRE and SWI [88]. However, PHIL 25% has reduced visibility as compared to Onyx 18 in small vessels because it is less radiopaque. PHIL™ 35% has achieved complete occlusion of aneurysms [89]. It has been proven to be safe and effective in the treatment of cranial dural arteriovenous fistulas, achieving complete embolization in most of cases [89]. PHIL 25% is more brittle and less pliable compared to Onyx [93,161]. Moderate vascular inflammation was observed with no angionecrosis in vessels filled with PHIL [93]. In contrast, reduced vascular inflammation was observed with Onyx-filled vessels, although angionecrosis was observed [159]. The smallest vessel reported occluded with PHIL 25% was 2.9 μm [160].

PHIL produces faster plug formation, which may decrease procedure time [93]. It also produces slower backflow, reducing the risk of unintended embolization of proximal vessels [88]. PHIL has an aesthetic advantage over Onyx for use in AVMs in the face due to its white color [93]. PHIL has been approved in Europe for use in arteriovenous malformations and hypervascular tumors [93]. PHIL has been used for treating type II endoleaks. In 2017, Helmy et al. reported that type II endoleaks of three patients were treated successfully using PHIL [162]. In 2022, Cheung et al. reported that the treatment of type II endoleaks was performed successfully with different combinations of PHIL 25%, 30%, and 35% [160].

In 2017, Vollherbst et al. performed a comparative study between ONYX and PHIL where an in vitro AVM model was embolized with these two embolic agents [10]. The results indicated that comparable embolization was achieved with lower volumes of PHIL than ONYX. Additionally, pause time had a considerable effect on the embolization success, shorter pauses resulted in better filling with PHIL. In 2022, Cheung et al. reported another comparative study between PHIL and Squid to investigate their efficacy and safety profile for the endovascular embolization of cerebral AVMs in twenty-three patients [163]. It was concluded that both the embolic agents were safe and effective, achieving satisfactory nidal obliteration rates and functional outcomes.

In 2018, Varadharajan et al. presented their preliminary experience using PHIL for the endovascular embolization of vascular shunts of eleven patients who had experienced satisfactory embolization results without recanalization for 6 months [164]. The authors have suggested further studies for evaluating safety and efficacy of PHIL. In a technical report in 2019, Kurda et al. demonstrated the treatment of uterine AVM by transcatheter uterine artery embolization using PHIL [165]. The authors have mentioned that the process was safe, effective and successful proving an alternative method for hysterectomy. In 2020, Lacis et al. reported an extremely rare case of parotid gland AVM, where it was treated by trans-arterial embolization using PHIL in a safe, feasible, and effective way [166]. In 2021, Lucatelli et al. reported the use of PHIL in non-neurological embolization procedures in thirty-five patients, where it was concluded that PHIL was a safe and effective embolic agent for the treatment of non-neurologic bleeding [167].

#### 3.2.5. EASYX^TM^

As early as 2006, iodine-containing polyvinyl alcohol systems have been investigated as liquid embolics for EE. Dudeck et al. evaluated the use of intrinsically radiopaque iodine containing polyvinyl alcohol as a liquid embolic in a swine model of side-wall, wide-neck aneurysms [168,169]. In 2016, Kulcsar et al. further investigated an iodinated polyvinyl alcohol, with the tradename EASYX, as a liquid embolic in two animal models [170]. These two animal models include swine rete mirabile as an AVM model and swine kidney as a hypervascular organ model. This study concluded that embolization with the iodinated polyvinyl alcohol liquid embolic is feasible and effective. Most recently, a multicenter prospective study evaluated the clinical safety and efficacy of EASYX for peripheral embolization [171].

## 4. Conclusions

This review article has discussed liquid embolic agents used for transcatheter endovascular embolization. A few liquid embolic agents, such as Eudragit-E, are currently no longer being used due to safety and feasibility issues. At present, a few precipitating liquid embolic agents, such as Onyx and Phil, are widely used for the purpose of endovascular embolization. Researchers are working to develop alternatives to these embolic agents, which are delivered with the undesirable, toxic DMSO solvent that can cause significant inflammatory response in patients. Furthermore, newer liquid embolic materials do not require toxic solvents such as DMSO which require special catheters for delivery. Therefore, several in situ gelling-type liquid embolic systems are being engineered and developed. Successful in vivo and pre-clinical studies show the promising future of these liquid embolic systems for endovascular embolization. These newer materials are also designed to be biocompatible with fewer imaging artifacts as compared to Onyx, and excitingly targeted to influence and allow regrowth of the endothelium over the aneurysmal orifice.

## Figures and Tables

**Table 1:** Liquid embolic materials discussed in this review.

Embolizing Agent	Trade Names	Ref(s).
N-butyl-2-cyanoacrylate (NBCA)	Histoacryl Avacryl Tru-Fill n-BCA	[2,3,4,27,28,29,30,31,32,33,34,35,36,37,38,39,40,41,42,43,44,45,46,47,48,49,50,51,52]
Fibrin Glue	Tisseel Hemaseel	[3,4,53,54,55,56,57,58,59,60,61,62,63,64]
N-isopropylacrylamide (NIPAAm) Copolymers		[4,65,66,67,68,69,70,71,72,73,74,75,76,77,78,79,80]
Poly(ethylene glycol)/Poly(propylene glycol)/Penta erythritol tetrakis (3-mercaptopropionate) (PPODA/PEGDA/QT)		[81,82,83,84,85,86,87]
Shear-Thinning Biomaterials		[88,89]
Silk Elastin Protein (SELP)		[90,91,92]
Poly(Ethylene Glycol) Hydrogel Embolization System (HES)	Instylla Embrace	[92,93,94,95,96]
Eudragit		[97,98,99,100]
Calcium Alginate		[101,102,103,104,105,106]
Ethylene vinyl alcohol copolymers	Squid and Squid Peri	[107,108,109,110,111,112,113,114,115,116,117]
	Onyx	[118,119,120,121,122,123,124,125,126,127,128,129,130,131,132,133,134,135,136,137,138,139,140,141,142,143,144,145,146,147,148,149,150,151,152,153,154,155,156,157,158]
Poly(lactide-co-glycolide) and poly(hydroxyethyl methacrylate)	PHIL	[159,160,161,162,163,164,165,166,167]
Iodinated polyvinyl alcohol	EASYX	[168,169,170,171]

## Data Availability

Not applicable.
